# Roles of mitochondrial fusion and fission in breast cancer progression: a systematic review

**DOI:** 10.1186/s12957-022-02799-5

**Published:** 2022-10-03

**Authors:** Jixiang Xing, Luyao Qi, Xiaofei Liu, Guangxi Shi, Xiaohui Sun, Yi Yang

**Affiliations:** 1 First College of Clinical Medicine, Shandong University of Traditional Chinese Medicine, Jinan, Shandong China; 2 The Seventh People’s Hospital Affiliated to Shanghai University of Traditional Chinese Medicine, Shanghai, 200137 China; 3 Department of Breast and Thyroid, Affiliated Hospital of Shandong University of Traditional Chinese Medicine, Jinan, Shandong China

**Keywords:** Breast cancer, Mitochondria, Fission, Fusion

## Abstract

**Background:**

Mitochondria play critical roles in cellular physiological activity as cellular organelles. Under extracellular stimulation, mitochondria undergo constant fusion and fission to meet different cellular demands. Mitochondrial dynamics, which are involved in mitochondrial fusion and fission, are regulated by specialized proteins and lipids, and their dysregulation causes human diseases, such as cancer. The advanced literature about the crucial role of mitochondrial dynamics in breast cancer is performed.

**Methods:**

All related studies were systematically searched through online databases (PubMed, Web of Science, and EMBASE) using keywords (e.g., breast cancer, mitochondrial, fission, and fusion), and these studies were then screened through the preset inclusion and exclusion criteria.

**Results:**

Eligible studies (*n* = 19) were evaluated and discussed in the systematic review. These advanced studies established the roles of mitochondrial fission and fusion of breast cancer in the metabolism, proliferation, survival, and metastasis. Importantly, the manipulating of mitochondrial dynamic is significant for the progresses of breast cancer.

**Conclusion:**

Understanding the mechanisms underlying mitochondrial fission and fusion during tumorigenesis is important for improving breast cancer treatments.

## Introduction

Breast cancer is the most common cancer and the second leading cause of cancer-related death in women [1]. Patients in the terminal stage usually exhibit tumor metastasis, which is fatal [2]. Breast cancer is heterogeneous and involves both genetic and environmental factors, and it can be divided into three groups according to the molecular and histological characteristics: hormone receptor (estrogen receptor (ER+) or progesterone receptor (PR+)), human epidermal receptor 2 (HER2+) and triple-negative (ER−, PR−, and HER2−) breast cancer. Thus, treatment should be based on the molecular characteristics of the cancer [3]. Although many studies have explored the molecular mechanisms underlying breast cancer, these mechanisms are still poorly understood; therefore, additional in-depth research studies and analyses are needed to identify more effective therapeutic approaches.

Mitochondria play crucial roles in a series of physiological processes, such as proliferation, differentiation, metabolism, and apoptosis [4]. Mitochondrial morphology is governed by the regulation of two antagonistic processes: fusion and fission [5]. Mitochondria can fuse with each other to form a larger network, and they may also split into smaller mitochondria [6]. Mitochondrial dynamics have been reported to play a critical role in the process of breast tumor metastasis [7]. The main proteins related to mitochondrial fission and fusion are members of the dynamin family, with fission mainly mediated by dynamin-related protein 1 (Drp1) and mitochondrial fission 1 (FIS1) and fusion mainly relying on mitofusin (Mfn1 and Mfn2) and optic atrophy 1 (OPA1) [8]. It has been reported that DRP1 phosphorylation or expression levels were involved in the regulation of mitochondrial fission and fusion [9]. Recently, increasing evidence has revealed that cancer development is closely related to dysregulated mitochondrial dynamics [10]. A number of reports have indicated that dysregulation of the mitochondrial network plays an important role in the invasion, metastasis, apoptosis, and autophagy of cancer cells [11, 12]. Furthermore, a previous study showed that mitochondrial fusion enhances OXPHOS while mitochondrial fission promotes glycolysis [13]. However, the critical roles of mitochondrial dynamics in the progression of breast cancer remain elusive. In this review, the regulatory mechanisms underlying mitochondrial fission and fusion in breast cancer and the role of mitochondrial dynamics in the proliferation, apoptosis, and metastasis of breast cancer cells are discussed to provide important insights for the development of new breast cancer therapy drugs that target mitochondria.

## Methods

### Search strategy

**Fig. 1:**
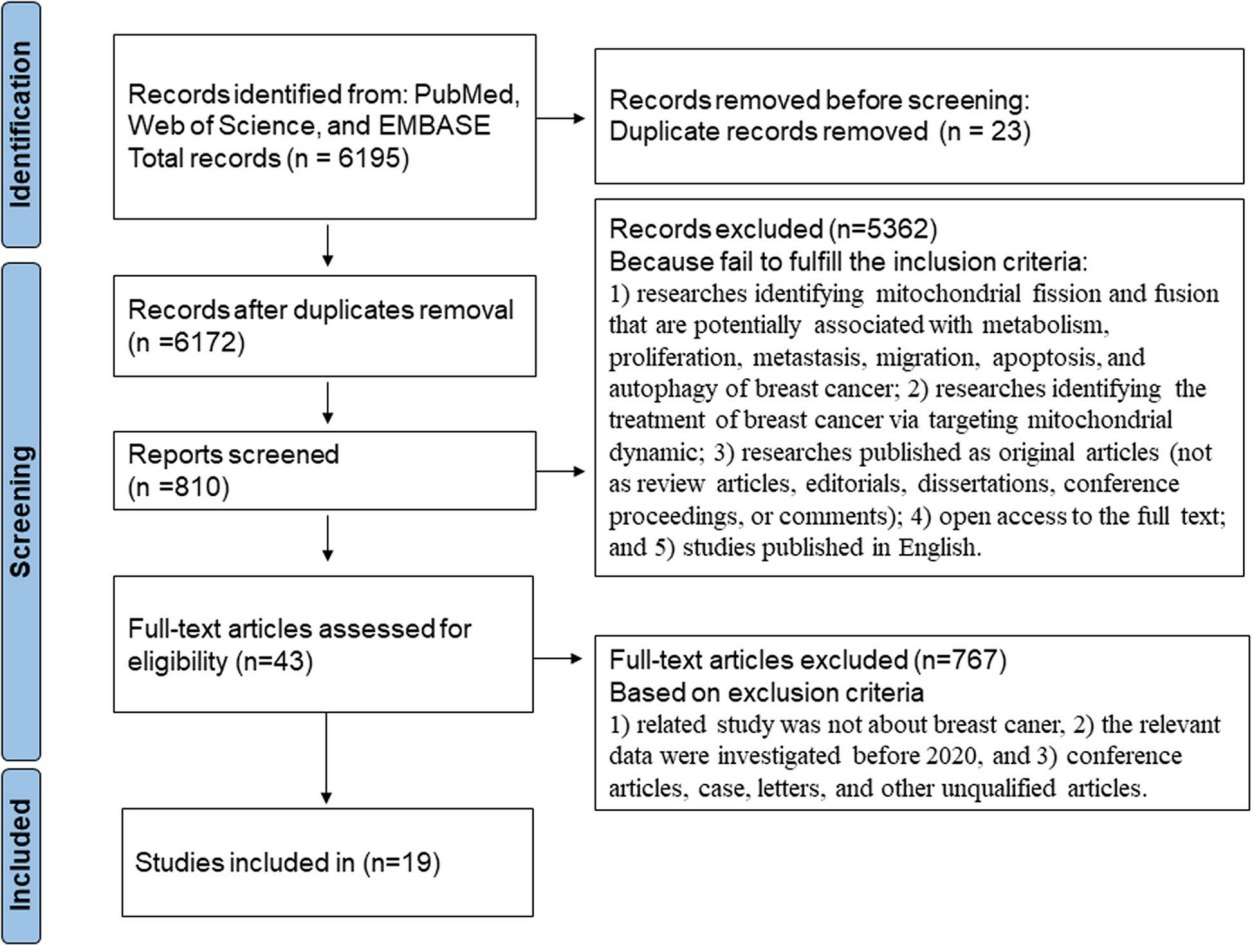
Study flow chart and selection process

We had systematically collected useful literature from PubMed, Web of Science, and EMBASE. Search terms included “breast cancer,” “mitochondrial,” “fusion,” “fission,” “metabolism,” “proliferation,” “metastasis,” “migration,” “apoptosis,” “autophagy,” and “treatment”. We expanded the range of the study by “related articles” option. A flowchart illustrating the study selection process and criteria for inclusion and exclusion is shown in Fig. 1.

### Eligibility criteria

The inclusion criteria were as follows: (1) researches identifying mitochondrial fission and fusion that are potentially associated with metabolism, proliferation, metastasis, migration, apoptosis, and autophagy of breast cancer; (2) researches identifying the treatment of breast cancer via targeting mitochondrial dynamic; (3) researches published as original articles (not as review articles, editorials, dissertations, conference proceedings, or comments); (4) open access to the full text; and (5) studies published in English.

Additionally, the exclusion criteria were as follows: (1) related study was not about breast cancer, (2) the relevant data were investigated before 2020, and (3) conference articles, case, letters, and other unqualified articles.

## Results

### Regulation of mitochondrial dynamics

Mitochondria are composed of an outer membrane, an inner membrane, an inner membrane space, and a matrix. The inner membrane consists of numerous cristae, and it is mainly involved in oxidative phosphorylation (OXPHOS). In the matrix, double-stranded mitochondrial DNA encodes 2 ribosomal RNAs, 13 protein electron transport chain (ETC) subunits, and 22 transfer RNAs [14]. Other mitochondrial proteins are encoded and transported from the nuclear genome. Mitochondria are essential for biosynthetic sensing and stress sensing to allow for cellular adaptations to various environments, especially the tumor microenvironment. Mitochondria have the ability to modulate their morphology after encountering various stimuli. Mitochondrial dynamics include morphological changes, intracellular distribution, and cytoskeletal transport. Under nutrient-deficient conditions, mitochondrial fusion occurs to promote the sharing of nutrient precursors and maintain OXPHOS. Smaller and fragmented mitochondria are derived from mitochondrial fission to fulfill the high energy demands of certain cell regions or perform mitosis [15]. The role of mitochondrial dysregulation in different cancers has been investigated for several decades, and the findings indicate that the mitochondrial architecture plays an important role during tumorigenesis.

**Fig. 2:**
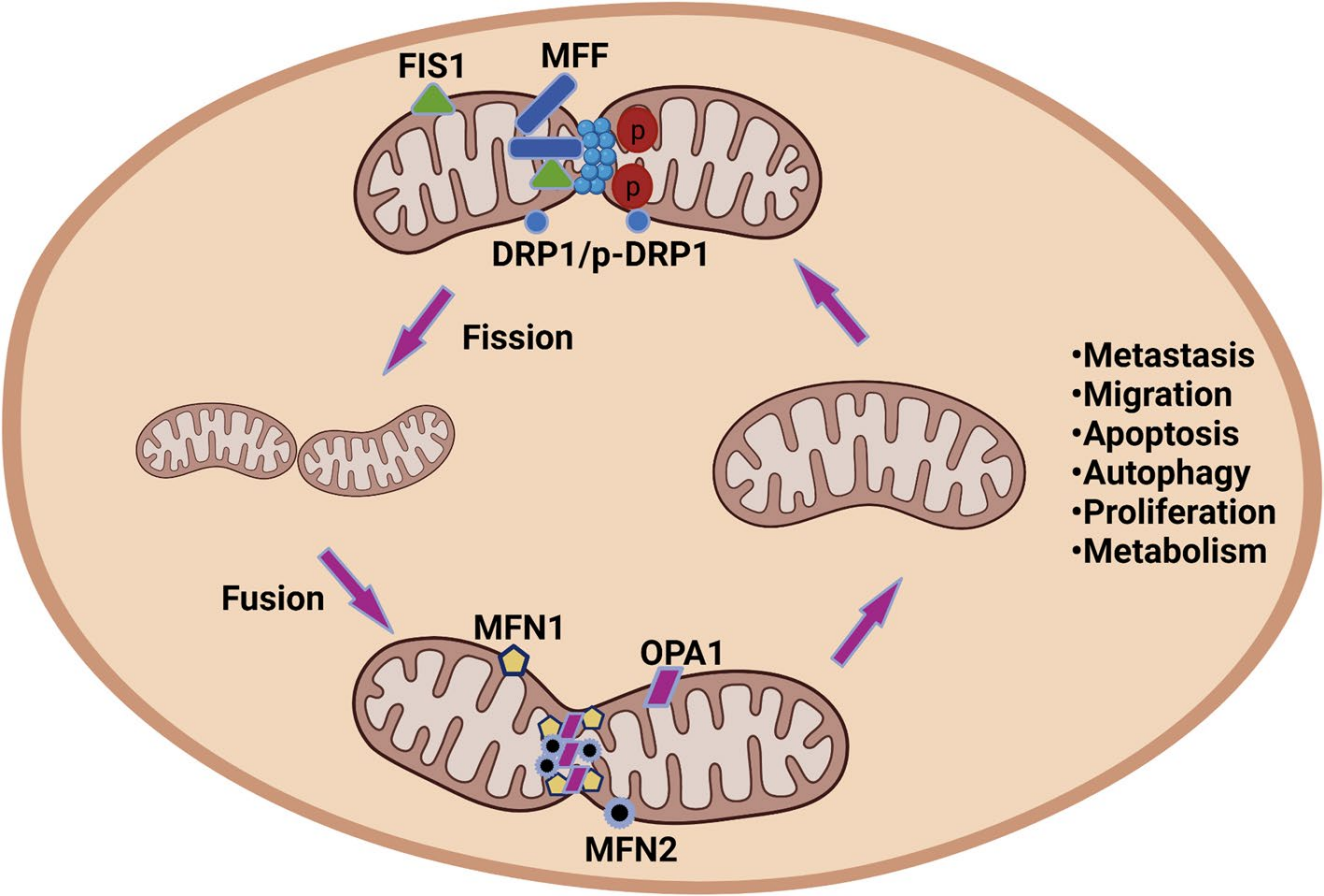
Mitochondrial fusion and fission mechanisms. This picture depicts a basic mechanism of mitochondrial fusion and fission, as well as significant factors that influence their regulation, which are discussed in greater depth in the main text

**Fig. 3:**
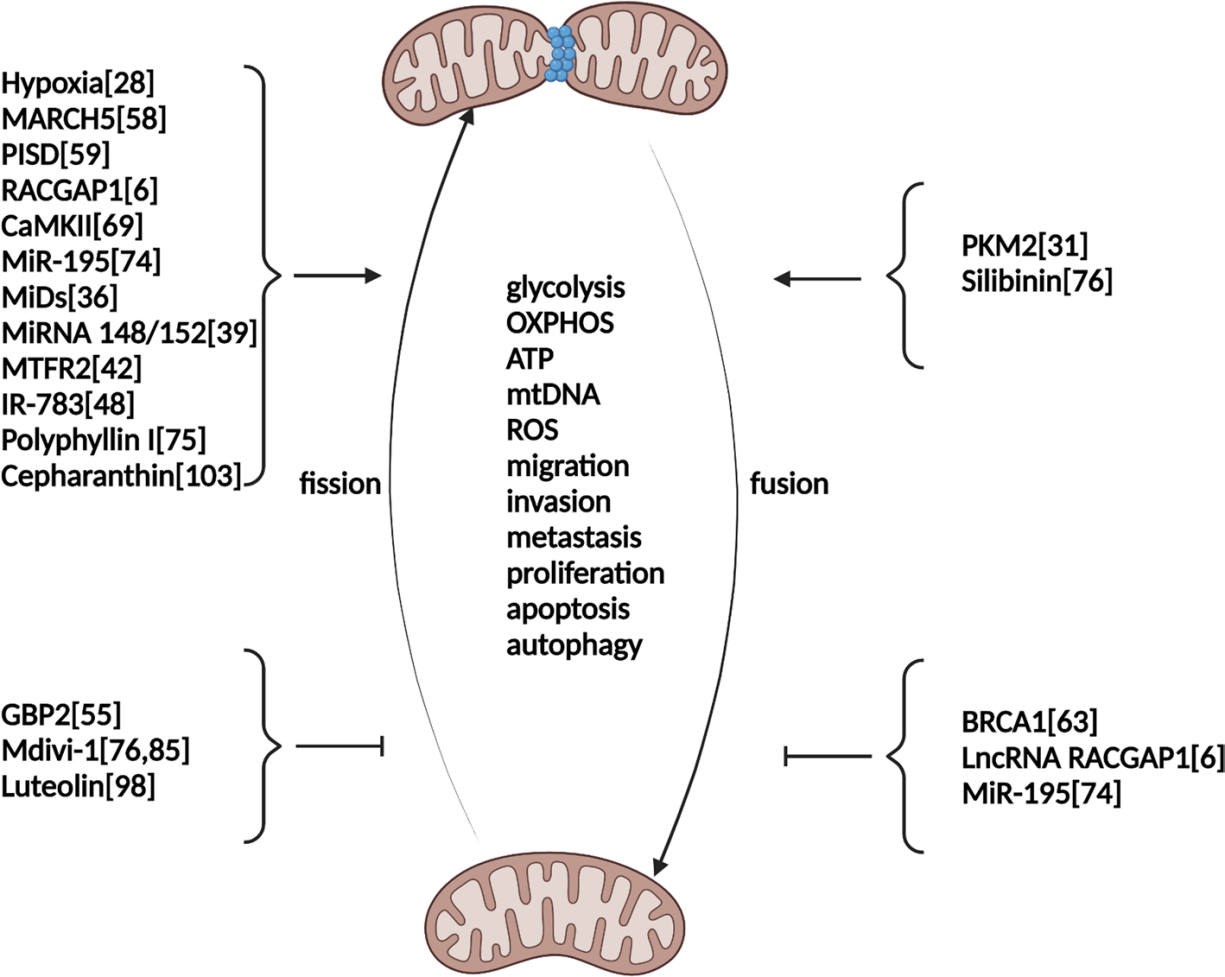
Regulators of mitochondrial fusion and fission in breast cancer. This picture depicts the roles of regulators in manipulating mitochondrial fusion and fission in breast cancer

The balance between mitochondrial fusion and fission is highly regulated to ensure the appropriate maintenance of mitochondrial functions. The regulation of mitochondrial fusion is mainly mediated by Mfn1, Mfn2, and OPA1. Mfn1 and Mfn2, which are involved in outer mitochondrial membrane fusion, are broadly expressed in a series of tissues [16]. Proteolytic ubiquitination is involved in the regulation of MFN1 and MFN2 via posttranscriptional modification [17]. OPA1 is located in the intermembrane space and is related to mitochondrial intermembrane fusion and cristae regulation [18]. The regulation of OPA1 expression mainly occurs via alternative splicing and proteolysis at the posttranscriptional level [19, 20]. In addition, mutations in OPA1 are involved in OXPHOS dysregulation, cristae morphology defects, and mtDNA instability [21, 22]. Mitochondrial fission is a hallmark of cancer and involved in the fragmentation, reorganization, and spatial distribution of mitochondria [23]. Mitochondrial fission is mediated mainly by the regulation of GTPase dynamin 1-like protein (DRP1), which is encoded by *DNM1L* [24, 25]. DRP1, which is located in the cytosol, regulates mitochondrial fission when recruited to the mitochondrial outer membrane, where it interacts with receptors. The role of DRP1 in mitochondrial fission is regulated by several posttranslational modifications, such as nitrosylation, ubiquitination, phosphorylation, and sumoylation [14] (Figs. 2 and 3).

### Mitochondrial dynamics in breast cancer metabolism

Metabolic reprogramming is a crucial step in tumorigenesis, and mitochondrial-mediated cellular metabolism plays a central role in cancer progression; however, the role of mitochondrial dynamics in metabolic regulation remains unclear. Reports have shown that increases in the demand for ATP associated with glycolysis and starvation could improve the OXPHOS ability of cancer cells [26]. Enhanced mitochondrial metabolism reprogramming is a critical characteristic of breast cancer stem cells (CSCs) during tumorigenesis, which reveals that targeting mitochondrial metabolism represents a potential method of inhibiting CSCs. MFF, located in the outer mitochondrial membrane, functions as the primary molecular regulator of mitochondrial fragmentation. Rosa et al. reported that MFF suppressed the mitochondrial biogenesis in MCF7 cells. In addition, the overexpression of MFF in breast cancer cells has a deleterious impact on the metabolic cell machinery, affecting OXPHOS, glycolysis, and CSC Activity, which indicates that improved mitochondrial fission may impair the metabolic processes required for breast cancer development [27]. Furthermore, they performed a proteomic analysis and found that the fatty acid metabolism pathway, oxidative stress-response pathway and hypoxia pathway were activated in MCF7 cells that overexpress MFF, which revealed that mitochondrial fission may impair breast cancer cell mitochondrial metabolism and could serve as a potential target for suppressing breast cancer progression [27]. A previous report indicated that PKM2 could induce mitochondrial dysfunction to reprogram mitochondrial metabolism from OXPHOS to glycolysis [28]. Furthermore, PKM2 overexpression promotes mitochondrial fusion by upregulating Mfn2, which decreases ATP levels and increases the mitochondrial DNA copy number [28]. Li et al. also reported that PKM2 could target MFN2 and improve mitochondrial fusion and OXPHOS in breast cancer [29]. Gao et al. found that salt-inducible kinase 2 (SIK2), a member of the AMP-activated protein kinase family, could enhance the Warburg effect of cancer by promoting mitochondrial fission [30]. Mechanistically, SIK2 could phosphorylate Drp1 at the Ser616 site and suppress the mitochondrial OXPHOS pathway, thus suggesting that mitochondrial fission mediated by the phosphorylation of Drp1 plays an important role in impairing the OXPHOS pathway and promoting cancer development [30]. These results indicate that mitochondrial fission and fission play crucial roles in metabolic reprogramming during breast cancer development.

The rigidity of the extracellular matrix (ECM) is a global signal that regulates cell proliferation, differentiation, and death. ECM rigidity is a key regulator of normal tissue function and contributes to disease-related cell behavior. The actin cytoskeleton and integrin-mediated adhesions help cells measure ECM rigidity by actively producing myosin-independent contractile forces. Recently, it was found that the mechanical cues in the ECM play a critical role in affecting mitochondrial metabolism. Romani et al. reported that mitochondrial fission was regulated by ECM rigidity via DRP1 [31]. The mitochondrial morphology of breast cancer cells grown on a soft ECM was changed. In breast cancer cells on a soft ECM, both endogenous DRP1 puncta and DRP1 phosphorylated at serine 616, which is an active form, were elevated. Importantly, DRP1 knockdown and induction with the DRP1 inhibitors MDIVI1 and Drpitor1a inhibited the generation of mtROS on a soft ECM. Furthermore, inhibiting DRP1 reduced cystine absorption and restored sensitivity to cumene hydroperoxide on a soft ECM. These findings revealed that soft ECM can activate DRP1 and cause mitochondrial fission and that DRP1 regulates metabolism by modulating mtROS and antioxidant capability in breast cancer cells (Table 1).

**Table 1:** Regulators of mitochondrial fission or fusion in breast cancer cells

Regulators	Cell line	Fission/fusion	Roles	References
Hypoxia	MDA-MB-231	Fission↑	Migration↑	[27]
GBP2	MDA-MB-231 and MDA-MB-436	Fission↓	Migration↓ and invasion↓	[32]
MARCH5	MDA-MB-231 and MDA-MB-468	Fission↑	Migration↑and metastasis↑	[33]
PISD	SUM159 and MDA-MB-231-PISD	Fission↑	Migration↑, OXPHOS↑	[34]
BRCA1	MDA-MB-231	Fusion↓	Migration↓, proliferation↓, and metastasis↓	[35]
RACGAP1/LncRNA RACGAP1	MCF7 and MDA-MB-231	Fission↑, Fusion↓	Migration↑, glycolysis↑, and metastasis↑	[6]
CaMKII	MDA-MB-231	Fission↑	Apoptosis↑ and ROS↑	[36]
MiR-195	MCF-7 and MDA-MB-231	Fission↑, Fusion↓	Apoptosis↑	[37]
Mdivi-1	MCF-7 and MDA-MB-231	Fission↓	Apoptosis↓, OXPHOS↓, ATP↓	[38, 39]
MiDs	MCF7	Fission↑ and Fusion↓	Proliferation↑and apoptosis↓	[40]
MiRNA 148/152	MDA-MB-231	Fission↑	Proliferation↑	[41]
MTFR2	Hs578T, MDA-MB-231, and MCF-7	Fission↑	Proliferation↑, invasion↑ and glycolysis↑	[42]
IR-783	MDA-MB-231 and MCF-7	Fission↑	Proliferation↓, migration↓, and ATP↓	[43]
PKM2	MCF-7	Fusion↑	mtDNA↑and ATP↓	[30]
Polyphyllin I	MCF-7 and MDA-MB-231	Fission↑	Apoptosis↑, cytochrome c↑, and mitophagy↑	[44]
Silibinin	MDA-MB-231	Fusion↑	Proliferation↓, migration↓, and invasion↓	[38]
Luteolin	4T1 and MDA-MB-231	Fission↓	Apoptosis↑ and metastasis↓	[45]
Cepharanthin	MDA-MB-231 and BT549	Fission↑	Apoptosis↑and autophagy↓	[46]

### Mitochondrial dynamics in breast cancer proliferation

The constant fusion and fission and dynamics of mitochondria are crucial for maintaining the physiological functions of cancer cells. It has been reported that increasing mitochondrial fission mediated by upregulated Drp1 improves the proliferation of hepatocellular carcinoma (HCC) cells mainly by promoting the G1/S phase transition of the cell cycle, which indicates that manipulating Drp1-dependent mitochondrial fission could be a potential method for suppressing HCC tumor growth [47]. Similarly, Yin et al. discovered that DRP1 expression was dramatically upregulated in glioma tissues compared to normal tissues and that inhibiting DRP1 expression suppressed cell proliferation and blocked pseudopodia formation [48]. Mechanistic studies further revealed that DRP1 could target RHOA, and thus regulate the proliferation and invasion of glioma cells.

In mammals, there are four Drp1 receptors on the mitochondrial membrane, including fission, mitochondrial 1 (Fis1), mitochondrial fission factor (MFF), mitochondrial elongation factor 2 (MIEF2, also known as MiD49), and mitochondrial elongation factor 1 (MEF1, also known as MiD51). Rehman et al. discovered that the upregulation of MiD49 and MiD51 improved mitochondrial fission in moderate hyperproliferative vasculopathy [49]. Recently, the roles of MiD49 and MiD51 were investigated in non-small-cell lung cancer (NSCLC) and breast cancer. Dasgupta et al. found that MiDs were pathologically upregulated in breast cancer cell lines, and in tumor tissues from breast cancer patients [40]. In addition, MiDs knockdown induced mitochondrial fusion and cell cycle arrest and thus inhibited proliferation by suppressing the activity of Drp1. Furthermore, suppressing the expression of MiDs attenuated tumor progression in a xenograft model. Mechanistic studies revealed that MiDs regulated mitochondrial fission via inhibiting the Akt-mTOR pathway and that dysregulated miR-34a-3p can target and manipulate MiDs in breast cancer cells.

OPA1, located in the inner mitochondrial membrane, plays an essential role in manipulating mitochondrial fusion and has been treated as a promising candidate in the area of directed mitochondrial cancer treatment. In addition, the dysregulated expression of OPA1 is involved in regulating cytochrome c release, cell proliferation and angiogenesis [50]. Furthermore, the OPA1 upregulation was linked to a poor prognosis, decreased chemotherapeutic sensitivity, and increased resistance to venetoclax [51]. Recently, the role of OPA1 in breast cancer was systematically investigated. Zamberlan et al. discovered that upregulated OPA1 expression is related to worse prognosis in breast cancer and that OPA1 downregulation can attenuate the proliferation, migration, and invasion of breast cancer [41]. Furthermore, they revealed that miRNA 148/152 can regulate the expression of OPA1 thus affecting the migration and proliferation in breast cancer cells.

In tumors, mitochondrial fission regulator 2 (MTFR2), known as family with sequence similarity, member A (FAM54A), has received little attention [52]. According to several studies, MTFR2 has a key role in mitochondrial and aerobic respiration, as well as promoting mitochondrial fission in cells [53]. Recently, the expression and physiological functions of MTFR2 in breast cancer were investigated. Lu et al. discovered that the expression of MTFR2 was increased in human breast cancer tissues and can be used as a prognostic diagnostic marker in patients [42]. Furthermore, they revealed that MTFR2 improved proliferation, invasion and aerobic glycolysis in breast cancer cells via regulating Hif1α and Hif2α.

Because of its well-visualized and significant tissue permeability qualities, near infrared (NIR) has a great potential in the detection and treatment of different diseases, such colorectal cancer, gastric cancer, and kidney cancer [54]. IR-783, as one of the NIR agents, has recently received much interest because of its remarkable imaging and tumor targeting characteristics. Several studies have previously shown that IR-783 can be selectively absorbed and enriched by cancer cells while causing minimal damage to normal organs [55]. Researchers have recently discovered that IR-783 can decrease the viability of cancer cells using pH-switchable photothermal therapy and photodynamic therapy [56]. IR-783 has also been shown to reduce MDA-MB-231 cell viability and increase mitochondrial apoptosis in human breast cancer cells, according to our earlier research [57]. A prior study reported that IR-783 can cause apoptosis in breast cancer cells by triggering mitochondrial fission [58]. Recently, the regulatory role of IR-783 in the proliferation and migration of breast cancer cells was investigated. Li et al. discovered that IR-783 can suppress the proliferation of MDA-MB-231 and MCF-7 cells via causing cell cycle arrest at the G0/G1 phase [43]. A mechanistic study further revealed that IR-783 induces mitochondrial fission by suppressing the expression of OPA1, Mfn1, and Mfn2, while enhancing the expression of MFF, Fis1, and Drp1. These findings revealed that IR-783 plays a critical role in attenuating the proliferation and migration of breast cancer cells and can be applied for the breast cancer treatment (Table 1).

### Mitochondrial dynamics in breast cancer metastasis and migration

The majority of breast cancer-related deaths are induced by metastasis, and approximately 50% of breast cancer patients will experience metastasis [59]. Zhao et al. reported that the mitochondrial fission-related protein Drp1 is upregulated significantly in invasive breast cancer and during metastasis to lymph nodes and upregulated to a greater degree in metastatic breast carcinoma than in non-metastatic cancer; moreover, the fragmented mitochondrial content was positively related to the expression of Drp1 [60]. Furthermore, Drp1 knockdown or Mfn1 overexpression induces mitochondrial elongation and significantly inhibits breast cancer cell metastasis, indicating that Drp1 can serve as a novel target for limiting breast cancer metastasis [60]. Hypoxia is a typical feature of the microenvironment of solid tumors, as well as a significant activator of migration and invasion. Hypoxia has been shown to affect cellular activity by stabilizing HIF-1α and HIF-1α, which are linked to distant metastasis and a poor prognosis, and upregulation is more common in breast cancer metastases than in the initial tumor [61]. Han et al. found that the expression of Drp1 was higher in metastatic MDA-MB-231 cells than in non-metastatic MCF-7 cells and hypoxia further increased Drp1 expression and induced mitochondrial fission in MDA-MB-231 cells but not in MCF-7 cells. In addition, hypoxia-stimulated mitochondrial fission and cell migration were considerably reduced in MDA-MB-231 cells when Drp1-dependent mitochondrial fission was inhibited by Mdivi1 or Drp1 knockdown, which confirmed the regulation of Drp1-mediated mitochondrial fission in hypoxia-induced breast cancer metastasis [62]. Similarly, hypoxia was reported to drive mitochondrial fission in GBM cells by upregulating Drp1 transcription and expression and Drp1 expression was prevented when HIF-1 was inhibited with echinomycin [63]. It has been documented that the upregulation of guanylate-binding protein 2 (GBP2), a GTPase, is linked to a better prognosis in breast cancer patients [64]. Zhang et al reported that GBP2 plays a crucial role in the suppressing mitochondrial fission and metastasis of breast cancer cells [32]. Mechanistically, GBP2 targets Drp1 and limits Drp1 translocation into mitochondria, thereby suppressing Drp1-dependent mitochondrial fission and breast cancer cell invasion, which suggests that GBP2 could be a potential therapeutic target for preventing breast cancer spread by inhibiting Drp1-dependent mitochondrial fission [32].

Membrane-associated ring finger 5 (MARCH5), is an E3 ubiquitin-protein ligase that regulates mitochondrial morphology by ubiquitinating DMN1L, MFN1, and MFN2 [65]. MARCH5 expression was significantly increased in BC cells, due to miR-30a downregulation, which contributed to poor survival in BC patients [66]. MARCH5 plays a critical role in increasing BC cell proliferation and metastasis in vitro and in vivo via promoting G1–S cell cycle arrest and epithelial–mesenchymal transition, which is mediated by facilitating mitochondrial fission and subsequent ROS generation [66]. Recently, MARCH5 was reported to trigger mitochondrial fission and mitophagy in response to ER stress by facilitating the ubiquitination and degradation of MFN2 in melanoma [33].

Phosphatidylserine decarboxylase (PISD), a mitochondrial enzyme that transforms phosphatidylserine (PS) to phosphatidylethanolamine (PE), has previously been shown to cause mitochondrial fission [34]. Recently, Humphries et al. reported that using genetic and pharmacological approaches to enforce mitochondrial fission lowered the metastatic potential of triple-negative breast cancer cells (TNBCs) [67]. Furthermore, they found that mitochondrial fission inhibited Akt and ERK signaling in cell and mouse models of breast cancer, and promoting mitochondrial fusion could reverse these characteristics and improve metastasis. In addition, higher expression levels of fission genes and lower expression of mitochondrial fusion genes were associated with increased survival of breast cancer patients, which suggests that promoting mitochondrial fission will be beneficial for breast cancer patients [67]. It has been reported that breast cancer susceptibility gene 1 (BRCA1), as a breast cancer suppressor gene, play a crucial role in keeping DNA intact and maintaining genomic stability [68]. In addition, TNBC accounts for more than 75% of cancers that arise in women with BRCA1 mutations. The nuclear location of BRCA1 has been linked to its tumor suppressor action, as it participates in DNA damage repair, cell cycle checkpoint control, and apoptosis signaling pathways [69]. Recently, Chen et al. revealed that BRCA1 involved in maintaining healthy mitochondrial network via regulating mitochondrial dynamics [35]. Furthermore, they disclosed that BRCA1 knockdown improved mitochondrial fusion by increasing the promoter activities of Mfn1 and Mfn2, thus upregulating the expression of MFN1/2. Increased BRCA1 on mitochondrial outer membranes was involved in activating AMPK pathways and promoting DRP1 recruitment to the mitochondrial outer membrane, which led to mitochondrial fission [35].

Rac GTPase activation protein 1 (RACGAP1) is a part of the central spindlin complex that functions as a microtubule-dependent and Rho-mediated signaling pathway for the production of myosin contractile rings during cell cycle cytokinesis [70]. The RacGAP1 gene is significantly upregulated in a variety of malignancies and is linked to a higher rate of tumor metastasis and a poor prognosis in breast cancer patients [71]. Recently, Ren et al. found that the upregulation of RACGAP1 in breast cancer cells could induce the fission of mitochondria. Furthermore, they showed that RACGAP1 improved mitochondrial fission via targeting ECT2 during anaphase and activating the ERK-DRP1 pathway, which promoted the metastatic progression of breast cancer [6]. Non-protein-coding transcripts more than 200 nucleotides in length are known as long non-coding RNAs (lncRNAs). A growing body of evidence has implicated that lncRNAs in the progression of human malignancies. LncRNA RACGAP1P, a member of the GTPase activating protein family, was reported to manipulate mitochondrial fission in the progression of breast cancer [69]. LncRNA RACGAP1P was found to be upregulated in breast cancer tissues and was linked to tumor metastasis and a poor prognosis in breast cancer patients. On the other hand, Mdiv1’s suppression of mitochondrial fission attenuated the invasion capacity of RACGAP1P-overexpressing breast cancer cells. Mechanistically, the enhancement of mitochondrial fission by RACGAP1P relied on its competitive targeting of miR-345-5p, causing the activation of Drp1, which revealed that lncRNA RACGAP1P facilitates breast cancer metastasis through miR-345-5p/RACGAP1 axis-mediated mitochondrial fission (Table 1).

### Mitochondrial dynamics in breast cancer apoptosis and autophagy

The interchange of proteins, mtDNA, and metabolites is facilitated by frequent fusion and fission, which helps to preserve mitochondrial integrity. Dysregulation of mitochondrial dynamic processes is also involved in impaired autophagy and apoptosis, thus affecting cancer progression. Recently, the role of Drp1 dysregulation in regulating mitophagy in breast cancer cells was investigated and inhibiting autophagy via targeting Drp1 could serve as a potential therapeutic strategy in cancer. Zou et al. found that Drp1 expression and mitochondrial biogenesis were upregulated and the mitochondrial number was decreased, which was linked to a reduction in mitochondrial oxidative ability in breast cancer cells [72]. Furthermore, they found that BNIP3, which plays a key role in mitochondrial autophagy, was also upregulated in breast cancer cells and that Drp1 inhibition significantly suppressed mitochondrial autophagy and decrease the viability of breast cancer cells, which revealed that Drp1 plays an essential role in regulating breast cancer cell survival via manipulating mitophagy. Mitochondrial fission and apoptosis are two interrelated events, and improved mitochondrial fission may represent a marker of apoptosis. CaMKII is a versatile serine/threonine protein kinase that regulates different physiological processes through the transmission of Ca2+ signals [73]. CaMKII has also been shown to regulate the phosphorylation of Drp1 at S616 and hence causes radiation-induced mitochondrial fission [74]. Recently, Hu et al. discovered that CaMKII promoted Drp1-dependent mitochondrial fission and apoptosis in TNBCs treated with isorhamnetin and chloroquine [36]. Moreover, they disclosed that mitochondrial fission and apoptosis were suppressed in cells overexpressing CaMKII and that the Thr286 phosphorylation was slightly upregulated in cells overexpressing CaMKII and that the Thr286 phosphorylation was activated under treatment with the isorhamnetin and chloroquine (CQ/IH) combination. Furthermore, CaMKII downregulation dramatically inhibited mitochondrial fission and apoptosis induced by CQ/IH, which suggested that CaMKII is needed for the Drp1-dependent mitochondrial fission and apoptosis.

Several investigations have shown that miR-195 is dysregulated in breast cancer tissues [75], and the enhanced methylation on the CpG island of the promoter of miR-195 is responsible for miR-195 downregulation in cancer tissue [76]. A prior study revealed that miR-195 is involved in promoting apoptosis in breast cancer and could attenuate the expression of Bcl2 via targeting its 3′UTR [77]. Another study showed that miR-195 exerts critical anti-proliferative, non-invasive, and anti-metastatic effects in breast cancer cells, which indicates that hsa-miR-195 has substantial anti-cancer activities and could serve as a target for breast cancer intervention [78]. In addition, miR-195 was also found to depolarize the inner membrane of mitochondria and raise calcium levels in mitochondria [78]. Recently, Purohit et al. reported that miR-195 could regulate mitochondrial dynamics and function and that the upregulation of miR-195 promoted mitochondrial fission [37]. They discovered that miR-195 improved mitochondrial fission by upregulating Drp1 expression and suppressing OPA1 expression, and the upregulation of miR-195 promoted the apoptosis of MCF-7 cells and MDAMB-231 cells by disrupting mitochondrial dynamics [37].

Li et al. reported that polyphyllin I, which is extracted from *Paris polyphylla* rhizomes, could dephosphorylate DRP1 and induce DRP1 mitochondrial translocation, thereby causing mitochondrial fission, cytochrome c release, and cell apoptosis. Mechanistically, DRP1 suppression could block polyphyllin I-induced mitochondrial fission and apoptosis [44]. Previously, Si et al. showed that silibinin could promote mitochondrial fission and suppress fusion and subsequent apoptosis in double-positive and triple-negative breast cancer cells. Furthermore, they reported that mdivi-1, an inhibitor of DRP1, could reverse silibinin-induced mitochondrial fission and that inhibiting mitochondrial fission via mdivi-1 could limit mitophagy in both MDA-MB-231 and MCF-7 cells [38].

A previous study showed that the activation of Drp1-mediated mitochondrial fission was linked to the release of cytochrome c and the induction of apoptosis [79]. However, further investigations revealed that Drp1 deficiency causes apoptosis to be delayed but not prevented, implying that mitochondrial fission is not required for apoptosis [80]. In addition, mitochondrial fission has been reported to improve the growth of breast cancer cells by suppressing apoptosis via a Notch-dependent pathway [72, 81]. Chang et al. reported that mitochondria transplantation into breast cancer cells could suppress Drp1 expression, promote mitochondrial fusion, and increase cell apoptosis [82]. In addition, they revealed that transplantation of Pep-1-conjugated mitochondria into mice bearing TNBC tumors promoted mitochondrial fusion and limited apoptosis, thereby suppressing tumor growth [83]. In summary, the above data suggest that mitochondrial fission might promote breast cancer progression, such as its initiation and development. However, excessive tumor microenvironmental stress could cause mitochondrial fragmentation and cell apoptosis.

It was recently discovered that ionizing radiation causes mitochondrial fragmentation by increasing Drp1-dependent fission in mouse fibroblasts [84]. Radiation-induced mitochondrial fragmentation was suppressed by Drp1 downregulation, as was radiation-induced clonogenic cell death and mitotic catastrophe, a type of cell death associated with aberrant mitosis, suggesting that radiation-induced mitochondrial fragmentation and its related function are partly linked to radiation-induced cell death [85]. Bo et al. reported that Drp1 appears to be marginally engaged in apoptosis, and that the suppression of mitochondrial fission reduces cellular radiosensitivity. Furthermore, it was discovered that mitotic catastrophe is a primary cause of radiation-induced cell death in EMT6 cells, and that radiation-induced mitotic catastrophe is mostly caused by mitochondrial fission [86]. They also disclosed that cytosolic [Ca2+], not ATP or ROS generation, is implicated in radiation-induced mitotic catastrophe, and that mitochondrial fission is partly engaged in mitotic catastrophe by raising cytosolic [Ca2+] following irradiation. These findings will favor the establishment of a strategy for modifying cellular radiosensitivity by intervening with mitochondrial dynamics (Table 1).

### Mitochondrial dynamic targeting in breast cancer

Recent studies have revealed that mitochondrial fission and fusion take part in oncogenesis and that mitochondrial dynamics could serve as a potential target for breast cancer treatment. Direct inhibition of fission can be achieved by blocking Drp1. Moreover, the mitochondrial division inhibitor Mdivi1 has been shown to selectively inhibit Drp1 and suppress mitochondrial fission in breast cancer cells [39]. Federico et al. found that Mdivi1 could inhibit ATP production and OXPHOS and promote apoptosis in breast cancer cells by suppressing mitochondrial fission [87]. In addition, Mdivi1 also serves as an inhibitor in several cancer cells, such as pancreatic cancer [88], HCC [89], gastric cancer [90], by manipulating mitochondrial dynamics. Han et al. also reported that Mdivi1 plays a crucial role in increasing the cisplatin sensitivity of ovarian cancer by suppressing mitochondrial fission [91]. However, the role of Mdivi1 in cancer progression has not been investigated in vivo. The regulators of mitochondrial fission and fusion can also serve as potential targets for breast cancer therapy. Silibinin is a polyphenolic flavonoid derived from *Silybum marianum* (milk thistle). It has been reported that silibinin could induce autophagy by causing mitochondrial malfunction and ATP depletion in human MCF7 breast cancer cells [92]. According to Byun et al., silibinin suppresses the Jak2/STAT3/MMP2 pathway, preventing MDA-MB-231 cells from proliferating, migrating, or invading [93]. Recently, Si et al. disclosed that silibinin treatment promoted mitochondrial fusion by suppressing the expression of DRP1 and enhancing the expression of Mfn1, Mfn2, and OPA1, leading to attenuated migration and invasion of breast cancer cells [94]. Additionally, silibinin plays an important role in other cancer cells such as colorectal cancer [95], ovarian cancer [96], and lung cancer [97] cells, and could serve as a potential therapeutic biomolecule. Doxorubicin (Dox) is a highly effective chemotherapy drug used to treat solid tumors and hematological malignancies. However, it induces dose-related cardiotoxicity in patients, which can lead to heart failure. In addition, Dox could cause mitochondrial fission via manipulating Drp1 expression [98]. Luteolin (Lut), a flavonoid found in a wide variety of plants, has been investigated for the treatment of a variety of disorders, including hypertension and cancer. Recently, Shi et al. documented that Lut could attenuate Dox-induced toxicity in cardiomyocytes [45]. Importantly, Lut effectively suppressed the improved mitochondrial fission after Dox treatment, thus weakening metastatic breast cancer. Lut also has anti-metastatic potential by targeting various signaling pathways associated with the suppression of epithelial to mesenchymal transition [99]. Jiang et al. reported that Lut could downregulate inducible PD-L1 expression to promote anti-tumor immunity in KRAS mutant lung cancer [100]. In addition, Lut is involved in the anti-inflammatory function in inflammatory bowel disease [101]. mDIVI1, a small molecule derived from quinazolinone, can selectively and reversibly target DRP1 [102]. mDIVI1 was demonstrated to regulate DRP1 by binding and decreasing both DRP1 self-assembly into ring-like structures and its ability to catalyze GTP hydrolysis in cancer stem cells, leading to the dysregulation of mitochondrial fission and suppressing cancer progression [39]. Autophagy inhibition has been identified as a viable cancer treatment method. Cepharanthin (CEP), a benzylisoquinoline alkaloid derived from Stephania cepharantha Hayata, is a natural anti-inflammatory and anti-tumor medicine that has been authorized for use in clinical trials to treat various acute and chronic disorders, including leukopenia, with minimal adverse effects [46]. Recently, Shen et al. found that CEP significantly suppressed cell survival and colony formation in human breast cancer MDA-MB-231 and BT549 cells. CEP and epirubicin work together to cause apoptosis in TNBC cells through the mitochondrial axis [103]. They also discovered that combining CEP and epirubicin increased Fis and Mff expression while decreasing Mfn1, Mfn2, and OPA1 expression. In addition, they reported that combining CEP and epirubicin causes mitochondrial fission and apoptosis and inhibits autophagy by inducing the dephosphorylation and translocation of Drp1. These results revealed that CEP inhibits autophagy/mitophagy and increases epirubicin-induced apoptosis in TNBC cells mainly by manipulating mitochondrial fission. In addition, CEP also suppressed endogenous ANO1 currents, significantly inhibited cell proliferation and migration, and induced death in lung cancer cells [104]. In addition, CEP could be used as a candidate drug for HCC that suppresses cell migration and proliferation [105]. IR-783, a near-infrared heptamethine cyanine dye, has a potential anti-cancer role. Recently, IR-783 was reported to inhibit the migration and proliferation of breast cancer cells by causing mitochondrial fission and subsequently suppressing ATP levels, leading to cell cycle arrest and inhibiting filopodia formation [43]. However, investigations of IR-783 in other cancers are lacking, and further research is needed (Table 1).

However, there are several challenges in treating breast cancer by targeting mitochondrial fission and fusion. Since tumor cells modify the mitochondrial rheostat in response to a range of stimuli to obtain proliferation and survival advantages, determining when stimulation or suppression of mitochondrial fission may help in the fight against tumor cells is a major challenge. In addition, it will be critical to determine whether changes in mitochondrial morphology are specific to different breast cancer mutations or to cancer cells in different tissues and whether mitochondrial dynamics may be targeted effectively for clinical the treatment of breast cancer.

## Conclusions

The study of mitochondrial dynamics in the breast cancer field is limited. However, growing evidence has reported the crucial role of mitochondria in metastasis, proliferation, apoptosis and metabolic reprogramming during cancer development. Understanding the regulatory mechanisms underlying mitochondrial dynamics is the basis of discovering novel targets for breast cancer therapy.

In this review, we summarized the significance of mitochondrial dynamics in biological processes associated with breast cancer. Elucidating the heterogeneity of the mitochondrial dynamics in different breast cancer cells and the mechanism of inducing mitochondrial dynamics are current problems that need to be solved. Additionally, determining whether activation or suppression of mitochondrial fission will work against tumor cells remains a challenge. Thus, in vitro and in vivo research is urgently required to discover potential pharmacological agents that manipulate mitochondrial dynamics in breast cancer. Additionally, the critical role of mitochondrial dynamics in stemness and chemotherapy resistance needs to be investigated because little information is currently available regarding breast cancer. Indeed, different tumors have different regulatory mechanisms. Do mitochondrial fission and fusion play the same role in different cancers? Or are mitochondrial fission and fusion regulatory mechanisms consistent across tumors? Do pharmaceuticals targeting mitochondrial fusion or fission in breast cancer work in other tumors or normal cells, and are the mechanisms of action the same? These issues deserve further exploration in the future.

In summary, studies on mitochondrial dynamics have revealed that fusion and fission are involved in breast cancer development; thus, more thorough investigations will be conducive to furthering our understanding of this disease and identifying better therapy options for breast cancer patients in the future.

## Data Availability

Not applicable.

## Abbreviations

ER: Estrogen receptor
PR: Progesterone receptor
HER2: Human epidermal receptor 2
FIS1: Fission 1
Mfn: Mitofusin
Drp1: Dynamin-related protein 1
OPA1: Optic atrophy 1
OXPHOS: Oxidative phosphorylation
ETC: Electron transport chain
GBP2: Guanylate-binding protein 2
TNBC: Triple-negative breast cancer
BRCA1: Breast cancer susceptibility gene 1
CSCs: Cancer stem cells
SIK2: Salt-inducible kinase 2
